# Correction: Children’s outdoor play at early learning and child care centres: Examining the impact of environmental play features on children’s play behaviour

**DOI:** 10.1371/journal.pone.0344301

**Published:** 2026-03-03

**Authors:** Rachel Ramsden, Ian Pike, Sally Thorne, Mariana Brussoni

In the Umbrella study subsection of the Materials and methods, there is an error in the third sentence of the paragraph. The correct sentence is: The ELCC centres involved in the PRO-ECO study were selected based on specific criteria: they were managed by the same organization (YMCA), served children aged 2.5 to 6 years, participated in the Affordable Child Care Benefit, were located near researchers at the University of British Columbia and were considered ready to join the study following informal interviews with the research team.

In the Inclusion/ exclusion criteria subsection of the Materials and methods, there is an error in the fifth sentence of the paragraph. The correct sentence is: Approval for this study was granted by the University of British Columbia (UBC) and the Children’s and Women’s Health Centre of British Columbia Research Ethics Board.

There is an error in reference 46. The correct reference is: Ramsden R, Han CS, Mount D, Loebach J, Cox A, Herrington S, et al. An Intervention to Increase Outdoor Play in Early Childhood Education Centers (PROmoting Early Childhood Outside): Protocol for a Pilot Wait-list Control Cluster Randomized Trial. JMIR Res Protoc. 2022;11(7):e38365. https://doi.org/10.2196/38365.

There is an error in reference 52. The correct reference is: Ramsden R, Mount D, Lin Y, Fox E, Herrington S, Loebach J, et al. Results from the PROmoting Early Childhood Outside cluster randomized trial evaluating an outdoor play intervention in early childhood education centres. Sci Rep. 2025;15(1):1713. https://doi.org/10.1038/s41598-025-85397-1.
