# Supplementary material for: Children’s outdoor play at early learning and child care centres: Examining the impact of environmental play features on children’s play behaviour
Source: PLoS One. 2025 Dec 10;20(12):e0318538. doi: 10.1371/journal.pone.0318538 (PMC12694797; doi:10.1371/journal.pone.0318538)
Supplement: S2 Appendix — (DOCX) [file pone.0318538.s002.docx]

# **S2 Appendix: Bivariate logistic regression results (OR, 95% CI) examining the association between each outcome and covariates**

**Table A1: Bivariate logistic regression results (OR, 95% CI) examining the association between play participation and environmental play features, topography, weather conditions, temperature, gender and loose part interaction.**

| **Variable** | **Bivariate Models – Physical Play** | | | | | |
| --- | --- | --- | --- | --- | --- | --- |
|  | Unadj. OR Model 1  [95% CI] | Unadj. OR Model 2  [95% CI] | Unadj. OR Model 3  [95% CI] | Unadj. OR Model 4  [95% CI] | Unadj. OR Model 5  [95% CI] | Unadj. OR Model 6  [95% CI] |
| **Environmental Play Feature** |  |  |  |  |  |  |
| Open Area | Ref |  |  |  |  |  |
| Gardening Area | 2.65 [1.75;4.03]*** |  |  |  |  |  |
| Fixed Equipment - Playhouse | 1.94 [1.37;2.75]*** |  |  |  |  |  |
| Fixed Equipment - Climbing Structure | 2.23 [1.68;2.96]*** |  |  |  |  |  |
| Sandbox | 2.55 [1.95;3.33]*** |  |  |  |  |  |
| Outdoor Stage | 2.21 [0.99;4.90] |  |  |  |  |  |
| Tricycle Path | 1.21 [1.01;1.46]* |  |  |  |  |  |
| Fixed Equipment - Water Feature | 5.36 [1.28;22.37]* |  |  |  |  |  |
| **Topography** |  |  |  |  |  |  |
| Low/ No Slope |  | Ref |  |  |  |  |
| Steep Slope |  | 3.69 [1.34;10.18]* |  |  |  |  |
| Uneven Terrain |  | 1.53 [1.16;2.00]** |  |  |  |  |
| **Weather Conditions** |  |  |  |  |  |  |
| Sunny |  |  | Ref |  |  |  |
| Cloudy, no rain |  |  | 0.93 [0.78;1.10] |  |  |  |
| Raining |  |  | 0.69 [0.55;0.86]*** |  |  |  |
| **Temperature** |  |  |  | 1.05 [1.03;1.07]* |  |  |
| **Gender** |  |  |  |  |  |  |
| Boy |  |  |  |  | Ref |  |
| Girl |  |  |  |  | 0.87 [0.76;1.00] |  |
| **Loose Part Interaction** |  |  |  |  |  |  |
| No |  |  |  |  |  | Ref |
| Yes |  |  |  |  |  | 2.74 [2.36;3.17]*** |

Unadj. = unadjusted; OR = odds ratio; 95% CI = 95% confidence interval

Note: significance level *: p<0.05, **: p<0.01, ***: p<0.001

**Table A2: Bivariate logistic regression results (OR, 95% CI) examining the association between physical play and environmental play features, topography, weather conditions, temperature, gender and loose part interaction.**

| Variable | Bivariate Models – Physical Play | | | | | |
| --- | --- | --- | --- | --- | --- | --- |
|  | Unadj. OR Model 1  [95% CI] | Unadj. OR Model 2  [95% CI] | Unadj. OR Model 3  [95% CI] | Unadj. OR Model 4  [95% CI] | Unadj. OR Model 5  [95% CI] | Unadj. OR Model 6  [95% CI] |
| **Environmental Play Feature** |  |  |  |  |  |  |
| Open Area | Ref |  |  |  |  |  |
| Gardening Area | 0.88 [0.67;1.14] |  |  |  |  |  |
| Fixed Equipment - Playhouse | 1.22 [0.95;1.58] |  |  |  |  |  |
| Fixed Equipment - Climbing Structure | 1.90 [1.54;2.36]*** |  |  |  |  |  |
| Sandbox | 1.42 [1.19;1.71]*** |  |  |  |  |  |
| Outdoor Stage | 1.67 [0.94;2.97] |  |  |  |  |  |
| Tricycle Path | 1.343[1.14;1.55]*** |  |  |  |  |  |
| Fixed Equipment - Water Feature | 1.14 [0.58;2.24] |  |  |  |  |  |
| **Topography** |  |  |  |  |  |  |
| Low/ No Slope |  | Ref |  |  |  |  |
| Steep Slope |  | 2.00 [1.12;3.58]* |  |  |  |  |
| Uneven Terrain |  | 1.00 [0.82;1.21] |  |  |  |  |
| **Weather Conditions** |  |  |  |  |  |  |
| Sunny |  |  | Ref |  |  |  |
| Cloudy, no rain |  |  | 0.94 [0.82;1.09] |  |  |  |
| Raining |  |  | 0.74 [0.61;0.88]** |  |  |  |
| **Temperature** |  |  |  | 1.01 [0.99;1.03] |  |  |
| **Gender** |  |  |  |  |  |  |
| Boy |  |  |  |  | Ref |  |
| Girl |  |  |  |  | 0.84 [0.75;0.94]** |  |
| **Loose Part Interaction** |  |  |  |  |  |  |
| No |  |  |  |  |  | Ref |
| Yes |  |  |  |  |  | 1.77 [1.56;2.01]*** |

Unadj. = unadjusted; OR = odds ratio; 95% CI = 95% confidence interval

Note: significance level *: p<0.05, **: p<0.01, ***: p<0.001

**Table A3: Bivariate logistic regression results (OR, 95% CI) examining the association between exploratory play and environmental play features, topography, weather conditions, temperature, gender and loose part interaction.**

|  | Bivariate Models – Exploratory Play | | | | | |
| --- | --- | --- | --- | --- | --- | --- |
| Variable | Unadj. OR Model 1  [95% CI] | Unadj. OR Model 2  [95% CI] | Unadj. OR Model 3  [95% CI] | Unadj. OR Model 4  [95% CI] | Unadj. OR Model 5  [95% CI] | Unadj. OR Model 6  [95% CI] |
| **Environmental Play Feature** |  |  |  |  |  |  |
| Open Area | Ref |  |  |  |  |  |
| Gardening Area | 1.39 [1.06;1.83]* |  |  |  |  |  |
| Fixed Equipment - Playhouse | 2.21 [1.72;2.83]*** |  |  |  |  |  |
| Fixed Equipment - Climbing Structure | 0.66 [0.53;0.83]*** |  |  |  |  |  |
| Sandbox | 4.77 [3.96;5.75]*** |  |  |  |  |  |
| Outdoor Stage | 3.33 [1.94;5.70]*** |  |  |  |  |  |
| Tricycle Path | 0.91 [0.77;1.07] |  |  |  |  |  |
| Fixed Equipment - Water Feature | 6.96 [3.26;14.86]*** |  |  |  |  |  |
| **Topography** |  |  |  |  |  |  |
| Low/ No Slope |  | Ref |  |  |  |  |
| Steep Slope |  | 0.43 [0.23;0.81]** |  |  |  |  |
| Uneven Terrain |  | 1.24 [1.02;1.51]* |  |  |  |  |
| **Weather Conditions** |  |  |  |  |  |  |
| Sunny |  |  | Ref |  |  |  |
| Cloudy, no rain |  |  | 0.98 [0.85;1.13] |  |  |  |
| Raining |  |  | 1.33 [1.11;1.60]** |  |  |  |
| **Temperature** |  |  |  | 1.05 [1.03;1.07] *** |  |  |
| **Gender** |  |  |  |  |  |  |
| Boy |  |  |  |  | Ref |  |
| Girl |  |  |  |  | 0.84 [0.75;0.94]** |  |
| **Loose Part Interaction** |  |  |  |  |  |  |
| No |  |  |  |  |  | Ref |
| Yes |  |  |  |  |  | 9.66 [7.80;11.97]*** |

Unadj. = unadjusted; OR = odds ratio; 95% CI = 95% confidence interval

Note: significance level *: p<0.05, **: p<0.01, ***: p<0.001

**Table A4: Bivariate logistic regression results (OR, 95% CI) examining the association between imaginative play and environmental play features, topography, weather conditions, temperature, gender and loose part interaction.**

|  | Bivariate Models – Imaginative Play | | | | | |
| --- | --- | --- | --- | --- | --- | --- |
| Variable | Unadj. OR Model 1  [95% CI] | Unadj. OR Model 2  [95% CI] | Unadj. OR Model 3  [95% CI] | Unadj. OR Model 4  [95% CI] | Unadj. OR Model 5  [95% CI] | Unadj. OR Model 6  [95% CI] |
| **Environmental Play Feature** |  |  |  |  |  |  |
| Open Area | Ref |  |  |  |  |  |
| Gardening Area | 1.19 [0.74;1.93] |  |  |  |  |  |
| Fixed Equipment - Playhouse | 1.61 [1.07;2.43]* |  |  |  |  |  |
| Fixed Equipment - Climbing Structure | 1.93 [1.42;2.62]*** |  |  |  |  |  |
| Sandbox | 1.19 [0.86;1.64] |  |  |  |  |  |
| Outdoor Stage | 1.91 [0.85;4.29] |  |  |  |  |  |
| Tricycle Path | 0.87 [0.64;1.19] |  |  |  |  |  |
| Fixed Equipment - Water Feature | 0.38 [0.05;2.80] |  |  |  |  |  |
| **Topography** |  |  |  |  |  |  |
| Low/ No Slope |  | Ref |  |  |  |  |
| Steep Slope |  | 1.24 [0.53;2.89] |  |  |  |  |
| Uneven Terrain |  | 0.88 [0.60;1.27] |  |  |  |  |
| **Weather Conditions** |  |  |  |  |  |  |
| Sunny |  |  | Ref |  |  |  |
| Cloudy, no rain |  |  | 1.02 [0.79;1.31] |  |  |  |
| Raining |  |  | 0.86 [0.61;1.21] |  |  |  |
| **Temperature** |  |  |  | 1.01 [0.98;1.05] |  |  |
| **Gender** |  |  |  |  |  |  |
| Boy |  |  |  |  | Ref |  |
| Girl |  |  |  |  | 1.13 [0.92;1.39] |  |
| **Loose Part Interaction** |  |  |  |  |  |  |
| No |  |  |  |  |  | Ref |
| Yes |  |  |  |  |  | 1.11 [0.87;1.41] |

Unadj. = unadjusted; OR = odds ratio; 95% CI = 95% confidence interval

Note: significance level *: p<0.05, **: p<0.01, ***: p<0.001

**Table A5: Bivariate logistic regression results (OR, 95% CI) examining the association between play with rules and environmental play features, topography, weather conditions, temperature, gender and loose part interaction.**

|  | Bivariate Models – Play with Rules | | | | | |
| --- | --- | --- | --- | --- | --- | --- |
| Variable | Unadj. OR Model 1  [95% CI] | Unadj. OR Model 2  [95% CI] | Unadj. OR Model 3  [95% CI] | Unadj. OR Model 4  [95% CI] | Unadj. OR Model 5  [95% CI] | Unadj. OR Model 6  [95% CI] |
| **Environmental Play Feature** |  |  |  |  |  |  |
| Open Area | Ref |  |  |  |  |  |
| Gardening Area | 0.87 [0.42;1.81] |  |  |  |  |  |
| Fixed Equipment - Playhouse | 0.09 [0.01;0.67]* |  |  |  |  |  |
| Fixed Equipment - Climbing Structure | 1.09 [0.67;1.78] |  |  |  |  |  |
| Sandbox | 0.21 [0.08;0.51]*** |  |  |  |  |  |
| Outdoor Stage | - |  |  |  |  |  |
| Tricycle Path | 1.61 [1.14;2.27]** |  |  |  |  |  |
| Fixed Equipment - Water Feature | - |  |  |  |  |  |
| **Topography** |  |  |  |  |  |  |
| Low/ No Slope |  | Ref |  |  |  |  |
| Steep Slope |  | 1.80 [0.65;5.02] |  |  |  |  |
| Uneven Terrain |  | 0.59 [0.31;1.12] |  |  |  |  |
| **Weather Conditions** |  |  |  |  |  |  |
| Sunny |  |  | Ref |  |  |  |
| Cloudy, no rain |  |  | 0.89 [0.63; 1.25] |  |  |  |
| Raining |  |  | 0.42 [0.23;0.75]** |  |  |  |
| **Temperature** |  |  |  | 0.98 [0.94;1.03] |  |  |
| **Gender** |  |  |  |  |  |  |
| Boy |  |  |  |  | Ref |  |
| Girl |  |  |  |  | 0.91 [0.67;1.22] |  |
| **Loose Part Interaction** |  |  |  |  |  |  |
| No |  |  |  |  |  | Ref |
| Yes |  |  |  |  |  | 0.24 [0.18;0.33]*** |

Unadj. = unadjusted; OR = odds ratio; 95% CI = 95% confidence interval

Note: significance level *: p<0.05, **: p<0.01, ***: p<0.001

**Table A6: Bivariate logistic regression results (OR, 95% CI) examining the association between bio play and environmental play features, topography, weather conditions, temperature, gender and loose part interaction.**

|  | Bivariate Models – Bio Play | | | | | |
| --- | --- | --- | --- | --- | --- | --- |
| Variable | Unadj. OR Model 1  [95% CI] | Unadj. OR Model 2  [95% CI] | Unadj. OR Model 3  [95% CI] | Unadj. OR Model 4  [95% CI] | Unadj. OR Model 5  [95% CI] | Unadj. OR Model 6  [95% CI] |
| **Environmental Play Feature** |  |  |  |  |  |  |
| Open Area | Ref |  |  |  |  |  |
| Gardening Area | 9.68 [6.83;13.73]*** |  |  |  |  |  |
| Fixed Equipment - Playhouse | - |  |  |  |  |  |
| Fixed Equipment - Climbing Structure | 0.27 [0.11;0.67]** |  |  |  |  |  |
| Sandbox | 0.43 [0.22;0.82]** |  |  |  |  |  |
| Outdoor Stage | - |  |  |  |  |  |
| Tricycle Path | 1.45 [1.02;2.07]* |  |  |  |  |  |
| Fixed Equipment - Water Feature | 0.75 [0.10;5.53] |  |  |  |  |  |
| **Topography** |  |  |  |  |  |  |
| Low/ No Slope |  | Ref |  |  |  |  |
| Steep Slope |  | 4.17 [1.95;8.90]*** |  |  |  |  |
| Uneven Terrain |  | 4.12 [3.00;5.66]*** |  |  |  |  |
| **Weather Conditions** |  |  |  |  |  |  |
| Sunny |  |  | Ref |  |  |  |
| Cloudy, no rain |  |  | 1.00 [0.72;1.37] |  |  |  |
| Raining |  |  | 0.56 [0.34;0.92]* |  |  |  |
| **Temperature** |  |  |  | 1.13 [1.08;1.18]*** |  |  |
| **Gender** |  |  |  |  |  |  |
| Boy |  |  |  |  | Ref |  |
| Girl |  |  |  |  | 1.13 [0.86;1.47] |  |
| **Loose Part Interaction** |  |  |  |  |  |  |
| No |  |  |  |  |  | Ref |
| Yes |  |  |  |  |  | 2.27 [1.53;3.37]*** |

Unadj. = unadjusted; OR = odds ratio; 95% CI = 95% confidence interval

Note: significance level *: p<0.05, **: p<0.01, ***: p<0.001

**Table A7: Bivariate logistic regression results (OR, 95% CI) examining the association between expressive play and environmental play features, topography, weather conditions, temperature, gender and loose part interaction.**

|  | Bivariate Models – Expressive Play | | | | | |
| --- | --- | --- | --- | --- | --- | --- |
| Variable | Unadj. OR Model 1  [95% CI] | Unadj. OR Model 2  [95% CI] | Unadj. OR Model 3  [95% CI] | Unadj. OR Model 4  [95% CI] | Unadj. OR Model 5  [95% CI] | Unadj. OR Model 6  [95% CI] |
| **Environmental Play Feature** |  |  |  |  |  |  |
| Open Area | Ref |  |  |  |  |  |
| Gardening Area | 0.74 [0.52;1.07] |  |  |  |  |  |
| Fixed Equipment - Playhouse | 1.03 [0.75;1.40] |  |  |  |  |  |
| Fixed Equipment - Climbing Structure | 0.85 [0.66;1.10] |  |  |  |  |  |
| Sandbox | 0.67 [0.52;0.85]** |  |  |  |  |  |
| Outdoor Stage | 0.80 [0.39;1.65] |  |  |  |  |  |
| Tricycle Path | 0.66 [0.53;0.81]*** |  |  |  |  |  |
| Fixed Equipment - Water Feature | 0.38 [0.12;1.25] |  |  |  |  |  |
| **Topography** |  |  |  |  |  |  |
| Low/ No Slope |  | Ref |  |  |  |  |
| Steep Slope |  | 0.79 [0.39;1.61] |  |  |  |  |
| Uneven Terrain |  | 0.79 [0.60;1.04] |  |  |  |  |
| **Weather Conditions** |  |  |  |  |  |  |
| Sunny |  |  | Ref |  |  |  |
| Cloudy, no rain |  |  | 1.08 [0.90;1.29] |  |  |  |
| Raining |  |  | 0.90 [0.70;1.15] |  |  |  |
| **Temperature** |  |  |  | 0.99 [0.97;1.01] |  |  |
| **Gender** |  |  |  |  |  |  |
| Boy |  |  |  |  | Ref |  |
| Girl |  |  |  |  | 1.18 [1.02;1.37]* |  |
| **Loose Part Interaction** |  |  |  |  |  |  |
| No |  |  |  |  |  | Ref |
| Yes |  |  |  |  |  | 0.79 [0.68;0.93]** |

Unadj. = unadjusted; OR = odds ratio; 95% CI = 95% confidence interval

Note: significance level *: p<0.05, **: p<0.01, ***: p<0.001
