# Supplementary material for: Children’s outdoor play at early learning and child care centres: Examining the impact of environmental play features on children’s play behaviour
Source: PLoS One. 2025 Dec 10;20(12):e0318538. doi: 10.1371/journal.pone.0318538 (PMC12694797; doi:10.1371/journal.pone.0318538)
Supplement: Attachment — Submitted filename: PLOS One - Response to Reviewers.docx [file pone.0318538.s004.docx]

**Rebuttal Letter to Editor and Reviewers**

Thank you for giving us an opportunity to revise and strengthen our paper. We have addressed all of the editorial and reviewer comments and questions – please see details in the table below.

**Editorial/Journal Requirement Comments**

| **Comment** | **Response** |
| --- | --- |
| Please ensure that your manuscript meets PLOS ONE's style requirements, including those for file naming. The PLOS ONE style templates can be found at https://journals.plos.org/plosone/s/file?id=wjVg/PLOSOne_formatting_sample_main_body.pdf and <https://journals.plos.org/plosone/s/file?id=ba62/PLOSOne_formatting_sample_title_authors_affiliations.pdf> | We have revised formatting throughout to meet all PLOS ONE style requirements. |
| Thank you for stating the following financial disclosure:  “This research was funded by the Lawson Foundation, grant GRT 2020-137.”  Please state what role the funders took in the study.  If the funders had no role, please state: "The funders had no role in study design, data collection and analysis, decision to publish, or preparation of the manuscript."  If this statement is not correct you must amend it as needed.  Please include this amended Role of Funder statement in your cover letter; we will change the online submission form on your behalf. | The funders had no role in study design, data collection and analysis, decision to publish, or preparation of the manuscript.  We have included this additional statement outlining the role of the funders in a revised cover letter with our revised submission. We appreciate you updating the online submission to include this information on our behalf. |
| 3. When completing the data availability statement of the submission form, you indicated that you will make your data available on acceptance. We strongly recommend all authors decide on a data sharing plan before acceptance, as the process can be lengthy and hold up publication timelines. Please note that, though access restrictions are acceptable now, your entire data will need to be made freely accessible if your manuscript is accepted for publication. This policy applies to all data except where public deposition would breach compliance with the protocol approved by your research ethics board. If you are unable to adhere to our open data policy, please kindly revise your statement to explain your reasoning and we will seek the editor's input on an exemption. Please be assured that, once you have provided your new statement, the assessment of your exemption will not hold up the peer review process. | We have begun the process of setting up a data repository for this study’s data on the UBC Research Data Collection (Borealis). |
| 4. Please include your full ethics statement in the ‘Methods’ section of your manuscript file. In your statement, please include the full name of the IRB or ethics committee who approved or waived your study, as well as whether or not you obtained informed written or verbal consent. If consent was waived for your study, please include this information in your statement as well. | We have included a statement on ethics approval and consent in the Methods section (lines 160-162). We redacted the approving ethics committee to ensure anonymity. |
| 5. Please include captions for your Supporting Information files at the end of your manuscript, and update any in-text citations to match accordingly. Please see our Supporting Information guidelines for more information: http://journals.plos.org/plosone/s/supporting-information. | We have added a new header/section at the end of our revised manuscript (lines 899-902) outlining the Supporting Information files and associated captions. In-text citations have been updated accordingly. We have also re-uploaded Supporting Information files with our revised submission to align with file naming directions. |

**External Peer-Review Comments – Reviewer 1**

| **Comment** | **Response** |
| --- | --- |
| Thank you for the opportunity to review this study. It reads well, but suffers from a lack of direction. The study questions are not teed up in the Introduction. The Intro should describe what is already known about associations between child play and the specific environmental features analyzed here so that by the time the reader reaches the measures and analytic strategy there are no surprises. Even in the Results, it is unclear exactly what question is being answered, and the second question seems to have disappeared completely. The Discussion is also a missed opportunity to highlight the novel insights provided by this study. I hope these comments allow the authors to significantly revise the manuscript to make it stronger. | Thank you for the thoughtful and informative comments. We appreciate the consideration and time taken to improve this paper. We have completed a major rewrite of the introduction to better prepare the reader to our research objective and associated analysis. We also clarified the research purpose and specific research question addressed in this paper. Lastly, we have reframed the discussion to better support the insights gathered in this paper. We present these updates further in each individual point below. |
| **Introduction** |  |
| p. 5, line 107: “While there is existing evidence on outdoor play environments that support children’s outdoor play, many studies have identified a gap in the research on the effects of specific natural and built design features and how they are used.” What is that gap? What specific natural and built design features are referred to here? The next paragraph on diversity of play does not seem to resolve these questions. | We have completed a major rewrite of the introduction to better outline the existing gap in the literature related to our research objective. |
| The authors write: “…Gibson’s theory of affordances supports the important question, “Are children using these spaces as intended?”” But you go on to write that children may view and use spaces in ways designers may not foresee. Isn’t that contradictory? So why is it important to ask that question? If the answer is yes, what does that tell us? And what does an answer of no tell us? Also, the rest of the paper seems to ignore this research question. | This is a great point. We have edited this statement to reflect our intended purpose and alignment with Gibson’s theory of affordances. In addition, we have removed the second research objective (related to intended vs. actual use) as we don’t feel our paper adequately addresses this in full. We have also further weaved Gibson’s theory of affordances, and how we applied this in the purpose of our study, into our Discussion. |
| By the time we get to “Research Purpose,” the study questions should be queued up in the reader’s mind, but they are not. Here, it says the first objective of the research is to analyze common environmental play features’ associations with children’s outdoor play. But what features exactly? What do we already know about those features’ associations with play? What outstanding questions does this study address? For example, are the descriptive statistics presented here on where play occurred novel or have other studies have conducted similar analysis? Just what does this study ask that others have not? | We have completed a major rewrite of the introduction to better prepare the reader to our research objective and associated analysis. We also clarified the research purpose and specific research question addressed in this paper. |
| And the second objective presented here – understanding the alignment between children’s use and designers’ intentions – should be justified in light of the questions I raise in my previous paragraph. And again, I would ask the authors to describe what is already known about this subject and what outstanding question is addressed here and how. | We have removed this second research objective as we don’t feel we can adequately address it within this paper (and it may fall into a future paper that incorporates additional data from this study). We have refined the Introduction to outline the existing evidence, a more clear research objective and the existing gap in the literature. |
| **Methods** |  |
| Why were these 8 particular sites chosen? | The selection of these child care centres is described in the study protocol for the larger study (referenced on line 153). We provided a brief description on lines 146-150. |
| How many coders were there? How were they certified as reliable? | In our original manuscript submission, we outlined the agreement level (k value of 0.918) that was achieved during training sessions prior to beginning data collection. We also outline how interrater reliability and agreement was assessed (lines 204-206). Similar to our response to the point above, further detail is outlined in the protocol paper (referenced on line 200). We have provided the number of coders on lines 210-212. |
| Line 238: “…additional rules were determined to categorize play observations that were coded as non-play or restorative play and another play type.” What were these rules and what were the codes? Why does that matter if you’ve already dichotomized behaviors into play vs. non-play? What were the final play variables? Counts based across episodes? | These rules are outlined in our primary results paper from the umbrella study (referenced on line 52). The rules and rationale for further dichotomizing this variable beyond the TOPO codes are quite detailed and we feel as though it would be too lengthy to provide the full description for this process (when it is already described elsewhere). However, we appreciate there may be confusion for a reader and have further clarified this process on line 225-231 and attached the rules in a new supporting information file (S1_Appendix). |
| Why were there 3 timepoints if the environmental play features didn’t change over time? Just to maximize the number of observations? If so, that’s fine, but say so. Specify which play features are considered fabricated and which are natural if this is an important distinction. | Yes, this study is part of a larger study (umbrella study: PRO-ECO) and data from all 3 data collection timepoints were used to maximize the number of observations. We have included this information on lines 152-153. We have added information on the manufactured nature of all examined elements in lines 242-243. |
| What is the “loose parts variable” mentioned in passing on line 254? | We have added a sentence on how this variable was collected and derived on lines 269-271. |
| What is the purpose of showing Figure 2 and Figure 3? What are observational behavior points (line 256)? Figure 3 needs a legend. | The purpose of Figure 2 and Figure 3 is to demonstrate the methodology used to define the Play Features variable (Figure 2) and provide an example of a base map with all OBM data points (Figure 3). These provide a snapshot of the methodology used and displayed on one of the participating centre’s base map. We felt it was relevant given the descriptive of our process may benefit from a visual representation.  Observational behaviour points represent a 15-second observations (collected through the OBM protocol) as outlined on line 195. On review, we realize this may be confusing as we also call these data points ‘play observations’. We have edited to reflect the use of ‘observational behaviour points’ throughout.  We have updated Figure 3 to include the legend. |
| If associations are being estimated between two things, they are bivariate, not univariate analyses (line 283). | Thank you – this was an error on our part in describing these analyses. We have changed the description to be ‘bivariate’ in the manuscript (Lines 276 & 290) and S2 – Appendix. |
| It is odd to read that the purpose of a multivariate logistic regression model is to obtain an “adjusted total effect.” Isn’t the purpose to estimate independent associations between play features and play? But more importantly, why didn’t the Introduction prepare the reader for this line of inquiry? | Thank you. This has been edited in the analysis section for clarity.  The introduction has been thoroughly revised to better prepare the reader for the analysis undertaken (and purpose). |
| The second research question presented in the Introduction seems to have disappeared. | This research question has been removed. We agree that this was not addressed within our analysis and distracts the reader from the primary goal of this paper. We have revised the Introduction accordingly. |
| How do models handle timepoint? How do they handle observations over time nested within centers? This is a critical oversight. | We used a Pooled Cross-Sectional Analysis approach by treating each time point as an independent cross-section and aggregating them to increase sample size *without* considering the temporal dimension (the structure of the data at each time point was assumed comparable). We did examine a model that included time to understand if the data differed or was impacted by the inclusion of time, including the possible interaction effect of time on the use of play features. Each data collection time point was treated as a discrete categorical variable. This supported examining differences between time points but it did not assume any linear/continuous trends. This process but did not find time, or an interaction with time, were significant to retain within the model. However, you raise a good point that it is not clear to the reader how time is handled and we have updated this within our methodology (lines 280-283).  To note, we also investigated this model within each time point and while the results were similar, the sample was often too small to adequately estimate each explanatory variable. |
| Why should centers with and without steep terrain be considered together, when they may have different associations between other environmental features and play? Same question for centers with and without loose parts. | The presence of steep and uneven terrain was very low across our participating centres. While all centres had uneven surfaces, these areas were only a small component of the total surface area. We have clarified and included this information in the Study sites section (lines 173-174). Based on this feedback, we re-ran our model with the inclusion of an interaction term examining play feature and topography together. This did not reveal any significant information, expect that there is a small significant negative effect on children’s play participation when a play structure is located on uneven topography. There was only one centre that had a play structure on uneven topography. This likely requires further investigation and we have recommended a future analysis examining the unique aspects of individual centres in our next steps (lines 607-609).  All included centres had loose parts and the opportunity to include loose parts in play - our data collection procedure recorded the presence of loose parts if a child interacted with it during an observational behaviour point. There were no significant interaction effects between loose parts and play features. |
| **Results** |  |
| Percentages should be reported as integers. | On review of the journal requirements and previous accepted papers, we noticed that there does not seem to be consistency on reporting percentages as integers or as a decimal. We have retained all percentages as is with decimals, based on our preference, but will check with the journal directly to confirm this is appropriate. |
| Tables 5 and 6 do not need unadjusted ORs; that information was already presented in Tables 3 and 4. The results regarding specific play types (Table 6) are too extensive for me to wade through. Because the Introduction did not prime me to attend to any particular dimension over any other, it is just too much information to process, which is why once again I return to the issue of what is important. If it is just a descriptive paper, all of the multivariate results are simply unnecessary. It may be sufficient to say, for example, that gardening areas are associated with children’s bio play without having to also claim that other types of features, much less unrelated factors like temperature, were controlled for. That may be a sufficient contribution to the literature, but I can’t tell based on the skimpy Introduction. | We have reorganized the presentation of the results based on your feedback. The primary analysis examined play vs. non-play, and Table 5 was meant to show the unadjusted effect in comparison to the adjusted effect. We retained Table 6 because we feel it is important to frame the results of this secondary analysis through a similar format as the primary analysis. While we have retained the unadjusted effects in Table 5 and 6, we did substantial edits to the Introduction, rationale for the study and the discussion to outline why these results (and this table) are important and noteworthy. |
| **Discussion** |  |
| The authors open this section by concluding that diverse outdoor play features offer more play participation for children than open areas. But all centers were examples of environments with diverse outdoor play features. Your coefficients only tell you about each specific feature relative to open area WITHIN a diverse outdoor environment. And was that the research question along? | The intention was to outline that most examined play features offered more forms of play to transpire, in comparison to open areas. We have edited this finding throughout to reflect our intended interpretation. |
| The next paragraph makes it seem like that the number of types of play is the outcome of interest, but the models did not address that question (there were no formal tests across play features by number of types of play). | Good point. Yes, you are correct in that our analysis wasn’t analyzing the number of forms of play associated with each environmental play feature, but rather an overview of which forms of play were associated with examined play features. These results contributed to discussing how this may contribute to diverse play experiences. We have revised this section, as well as the Introduction and research objectives, to provide clarity related to this confusion. |
| The authors should consider the possibility of making use of the variation among the 8 sites – rather than simply combining them all – to answer questions about how particular features work in the presence or absence of other features – again, if that is indeed their question. | The analysis focused on individual play features in the environment, rather than how these features were integrated with one-another. With eight participating centres, it was challenging to look at each centre individually in one paper to examine all the possible differences or unique results by site. Since this data is from a larger study, we anticipate that there will be site-specific analyses conducted in the future. We do note that the research objective was not clear in the original manuscript, and we have revised the research objective to further clarify our decision to look at all centres collectively. Mainly, all centres were operated by the same organization and contained similar play features, supporting looking at these environments collectively. We plan to do further analyses, including incorporating qualitative data from the larger study, on the differences by site in the future. We have included this as a recommendation for further analyses (lines 607-609). |
| By the end of this manuscript, I’m still not sure what specific knowledge gap this study addresses. | We have completed a major rewrite of the introduction and discussion sections to clarify the purpose of the stud, the existing knowledge gap and how this study fills this gap. |

**External Peer-Review Comments – Reviewer 2**

| **Comment** | **Response** |
| --- | --- |
| Thank you for submitting the manuscript titled "Children’s Outdoor Play at Early Learning and Child Care Centres: Examining the Impact of Environmental Play Features on Children’s Play Behaviour." The paper applies Gibson’s theory of affordances to explore the relationship between children’s outdoor play spaces and their behavioural patterns, which is a highly relevant topic. It effectively summarizes the current evidence and identifies an important gap in the existing knowledge. | Thank you for your detailed comments and contribution to improve this paper. |
| Overall, I suggest updating the literature review. While many references are cited, I believe that including more current sources would strengthen the manuscript. For example, the latest special issue from AJOT (https://research.aota.org/ajot/issue/78/4) and other recent works could provide valuable insights. | We have updated the Introduction section considerably to further define our research objective, the existing knowledge and provided updated sources. We appreciate the link and direction towards the recent AJOT work. |
| While the use of observations and behavioural mapping is a child-friendly method, I would recommend considering a more holistic approach that centers on children’s own experiences and reflections. This would provide valuable insight into their reasoning and decision-making during play. Such an approach would complement the affordances theory proposed by Refshauge, which presents a more "able" perspective, yet also has its limitations. This theory is largely guided by an adult’s perspective on what constitutes, for example, "jump-on-able" or "balance-able" features. However, from a more dynamic standpoint, children with diverse abilities may identify and engage with these features differently. I believe it would be beneficial to mention children with varying abilities as key users of outdoor spaces to highlight the wide range of possible occupations and experiences they can have. | Thank you for this feedback. We agree that the observations and use of behaviour mapping has its limitations, most notably, the reliance on adults (us as researchers) to make assumptions and interpretations that may not reflect children’s experiences and thoughts. To address this, we collected children’s qualitative perspectives of outdoor affordances as part of the umbrella study this analysis is situated in. These results will be presented in a separate paper (hopefully also this year). In the future, we hope to integrate these two data pieces to provide a more holistic approach to understand outdoor play affordances. We’ve bolstered the limitations to address this important point and provided further information on next steps of this research work to provide this context to the reader.  This analysis did not specifically consider the unique and varying abilities of children, however, it is an important consideration related to outdoor play affordances and we have added a sentence in the discussion to acknowledge this (lines 616-618). |
| Additionally, I find that the manuscript is quite long, and some results are discussed in the discussion section but not explicitly presented in the results section. I suggest synthesizing and condensing the manuscript to make it more concise and focused. | We have revised the Introduction and Discussion sections considerably to address both reviewer’s comments. In this process, we aimed to also condense the manuscript, in particular the Discussion and Results to be more focused on our research objective. |
| Overall, I believe this is an interesting and important manuscript. The methodology employed is novel within this field and makes a valuable contribution to the existing body of knowledge. | Thank you. |
